# Therapeutic strategy for incarcerated obturator hernia: laparoscopic surgery after ultrasound-guided noninvasive manual reduction

**DOI:** 10.3389/fsurg.2026.1835718

**Published:** 2026-05-20

**Authors:** Yoshihiro Shioi, Toma Kawashima, Hideki Kumagai, Kohei Ito, Mao Tsushima, Yamato Fujii

**Affiliations:** 1 Department of Surgery, Iwate Prefectural Senmaya Hospital, Ichinoseki, Iwate, Japan; 2 Department of Surgery, JA Akita Kouseiren, Kazuno Kousei Hospital, Kazuno, Akita, Japan; 3 Department of Surgery, Iwate Medical University, Morioka, Iwate, Japan; 4 Department of Surgery, Iwate Prefectural Daito Hospital, Ichinoseki, Iwate, Japan

**Keywords:** laparoscopic surgery, manual reduction, obturator hernia, plug repair, ultrasound-guided

## Abstract

**Background:**

Obturator hernia is a rare but serious disease associated with high morbidity and mortality, as it usually develops in the elderly. Herein, we evaluated the results of a less-invasive strategy for incarcerated obturator hernia (IOH) treated by laparoscopic repair after ultrasound sonography reduction. Study design: We retrospectively reviewed the records of 15 consecutive patients with IOH treated between April 2019 and March 2025.

**Results:**

Fifteen patients with IOH were treated at our hospital. Of these, 13 patients underwent ultrasound-guided, non-invasive manual reduction. One patient underwent emergency laparotomy due to suspected small bowel necrosis, and one patient underwent emergency laparoscopic surgery due to unsuccessful reduction. Elective laparoscopic surgery was attempted in 12 patients, of whom 10 patients underwent laparoscopic mesh-plug hernioplasty. One patient developed bowel necrosis and perforation after manual reduction and subsequently underwent laparoscopic surgery with simple ligation. One patient required conversion to open surgery due to intraperitoneal adhesions. One patient died of age-related causes without undergoing surgery after manual reduction.

**Conclusions:**

Laparoscopic repair after ultrasound-guided manual reduction for IOH is an effective and minimally invasive therapeutic strategy. However, patients with IOH are often elderly and frail, with multiple comorbidities; moreover, the small intestine may become incarcerated, leading to necrosis and perforation. Therefore, early diagnosis and intervention are essential. Additionally, continuous and careful evaluation of the patient's overall condition throughout treatment is necessary.

## 1 Introduction

An obturator hernia is an acute abdominal wall hernia that most commonly affects older, thin women and is characterized by intestinal prolapse through the pelvic gap (obturator foramen). Diagnosis is often delayed because external bulging is absent, which frequently results in intestinal obstruction or necrosis. Therefore, early imaging diagnosis using computed tomography (CT) or similar modalities is essential. The rarity of an obturator hernia (0.05%–1.4% of all abdominal wall hernias) limits the variability of treatment strategies and disease management across institutions (1). Furthermore, because pelvic hernias often present acutely, it is necessary to employ methods to relieve intestinal obstruction and assess intestinal ischemia. Recently, the number of cases wherein abrupt release has been achieved via ultrasonography-guided body surface compression has increased. Obturator hernia incarceration is common among elderly women, who frequently present with comorbidities. Therefore, less invasive and more definitive treatment approaches for this condition are desirable.

This study adopts a minimally invasive treatment policy for patients with incarcerated obturator hernia (IOH), involving manual hernia reduction when feasible, followed by elective laparoscopic surgery. Fifteen cases treated at a single facility are reported.

## 2 Material and method

### 2.1 Clinical data

We compiled medical records of 15 patients with IOH treated at our hospital between April 2019 and March 2025. The study protocol was approved by the ethics committee of Iwate Prefectural Senmaya Hospital. The diagnosis, manual reduction, laparoscopic surgery, and postoperative management of IOHs at this hospital were conducted under the supervision of a single experienced board-certified surgeon specializing in gastroenterology.

IOHs were diagnosed using contrast-enhanced CT scans. For patients without evidence of intestinal necrosis, ultrasound confirmed intestinal protrusion through the obturator foramen. The protruding intestine was then compressed with the thumb and manually reduced (2). Emergency surgery was conducted on patients who were determined to have intestinal necrosis. Patients who successfully underwent manual reduction subsequently received elective laparoscopic surgery. The laparoscopic approach for IOH utilized the surgical procedure previously described by Kawashima et al. (3). Patients for whom manual reduction was unsuccessful proceeded to emergency surgical intervention. The duration of patients' follow-up extended from April 2019 to March 2025. A limitation of this study is that the small sample size precluded formal statistical comparisons.

## 3 Results

Characteristics of the patients diagnosed with IOH are shown in Table 1. A total of 15 patients underwent treatment at our hospital for acute IOH. All of them were female and elderly patients, with a median age of 91 years. No significant difference was detected between the right and left sides. The median body mass index (BMI) of the cohort was 17.2, indicating that most patients were underweight. Within the ASA classification, category II represented 66% of cases. Six patients had a history of prior abdominal surgery, and 40% of patients were receiving antithrombotic drugs.

**Table 1:** Characteristics of the patients diagnosed as incarcerated obturator hernia.

Characteristics	Result
Sex (male: female)	0: 15
Age, median (range), years	91 (70–95)
Location (right: left)	8: 7
BMI, median (range), kg/m^2^	17.2 (12.9–23.8)
ASA-PS (II: III: IV)	10: 4: 1
Prior surgical procedures in abdomen (absent: present)	9: 6
Antithrombotic drugs (absent: present)	9: 6
Intestinal obstruction (absent: present)	0: 15
Duration from symptom onset to reduction, median (range), h	7.5 (4–168)
Echo-guided reduction (successful: unsuccessful)	13: 2
Duration from reduction to surgery, median (range), days	4 (0–9)
Operation (no operation: open: lap→open: lap)	1: 1: 1: 12
Small bowel perforation (absent: present)	12: 2
Operation methods (open-mesh: lap-plug: simple ligation)	1: 11: 2
Surgical time, median (range), min	57.5 (34–122)
Blood loss, median (range), g	3 (1–6)
Postoperative hospitalization, median (range), days	9 (3–61)
Complication (none: WH: IPH: peritonitis: CI)	10: 1: 1: 1: 1
Recurrence (absent: present)	15: 0
Hernia at other sites (absent: present)	11: 3

WH, wound hematoma; IPH, intrapelvic hematoma; CI, cerebral infarction; BMI, body mass index; ASA-PS, american society of anesthesiologists-physical status; LAP, laparoscopic surgery; Open, open surgery.

In every instance, intestinal obstruction was identified using CT. The duration from onset to recovery ranged from 4 to 168 h, with a median of 7.5 h, indicating a comparatively brief period for diagnosis. Manual reduction under ultrasound guidance was successful in 13 of 15 cases, yielding a reduction rate of 86.7%. In two cases where reduction was not feasible, intestinal necrosis was suspected in one instance, necessitating the removal of the necrotic intestine via emergency laparotomy without reduction. In the other case, emergency laparoscopic surgery was performed, and intestinal reduction was achieved intraoperatively. Among the 14 surgical cases, laparoscopic surgery was attempted in 13 cases, including one emergency procedure, and was successfully completed in 12 (85.7%). One case required conversion to open surgery due to intraperitoneal adhesions. Among the cases treated laparoscopically, 11 underwent repair using a plug. In one case involving intestinal perforation, the perforation site was sutured, and the obturator hernia was closed with a simple ligation. One patient underwent successful reduction but did not receive surgical intervention due to advanced age and died 17 days after admission. Intestinal perforation occurred in two of the 15 cases (13.3%). In both instances, more than 96 h had elapsed since symptom onset. Among the 14 surgical cases, plug repair was performed in 11 cases without perforation, while a simple ligation was utilized in two cases with perforation. Surgical times ranged from 34 to 122 min, with a median duration of 57 min. Blood loss was minimal across all procedures. The duration of postoperative hospitalization ranged from 3 to 61 days, with a median of 9 days. Among the 14 patients who underwent surgery, ten experienced no postoperative complications. One patient developed cerebral infarction, one had wound bleeding, one had a pelvic hematoma, and one developed peritonitis following perforation. Postoperative recurrence was not observed in any patient. Furthermore, antithrombotic drugs for cardiac or cerebrovascular conditions were administered in six of the 15 cases and were maintained throughout the treatment period.

**Figure 1:**
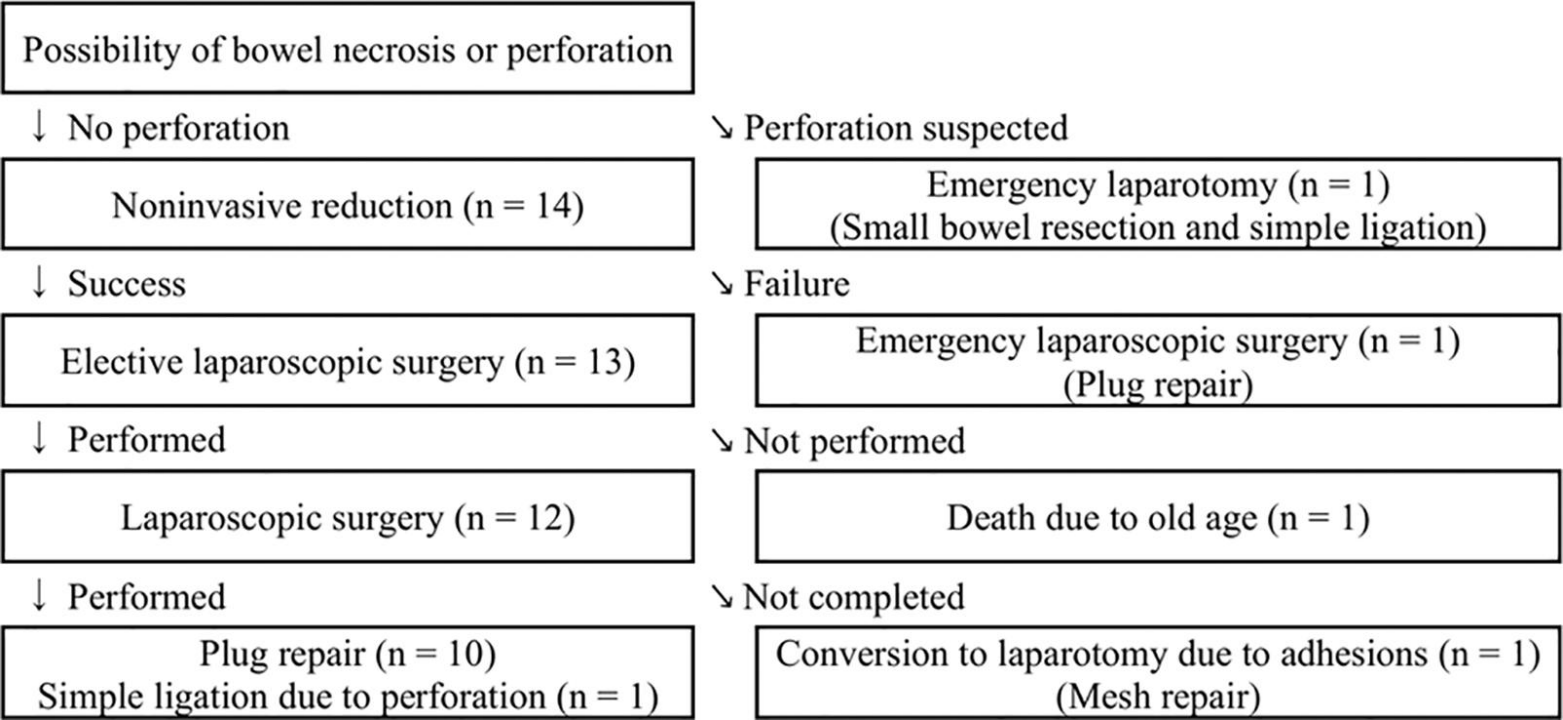
Flow chart depicting the management of incarcerated obturator hernia (*n* = 15).

The flow chart of the surgical strategy is shown in Figure 1.

## 4 Discussion

Obturator hernia should be included in the differential diagnosis of intestinal obstruction in elderly women. Obturator hernias result in acute intestinal obstruction and intestinal ischemia, primarily because the small intestine becomes trapped within the obturator foramen. Therefore, prompt diagnosis and intervention are essential. Furthermore, as the condition is more prevalent among elderly individuals with multiple comorbidities, a minimally invasive treatment strategy is warranted. Therefore, we employed laparoscopic surgery following noninvasive manual reduction.

Noninvasive reduction may be performed manually or with ultrasound guidance. The method employed at the hospital involves confirming the prolapsed intestine under ultrasound guidance, followed by manual reduction through compression with the thumb (2, 3). The success of this non-invasive reduction depends on practitioner expertise, with reported success rates across facilities ranging from 42% to 94% (4–6). At our institution, reduction procedures were conducted under the supervision of a single experienced gastroenterological surgeon, achieving a success rate of 86.7%. Accumulating experience in manual reduction remains essential.

One important limitation of this study is that all manual reductions were performed under the supervision of a single, highly experienced surgeon. While this ensured internal consistency within our cohort, it introduced a potential operator-dependent bias that may limit the reproducibility of our outcomes. The reported success rates and safety profiles therefore reflect the clinical judgment and technical proficiency of an individual, which may not be directly generalizable to clinicians with varying levels of experience or to other institutional settings. To establish the broader clinical utility of this technique, further multicenter studies involving multiple operators are required.

Successful manual reduction has three main benefits.

First, manual reduction may be used to stabilize the patient's condition before proceeding with elective surgery. Elective surgery is associated with reduced hospital stays and lower 30-day mortality rates (7). In emergency surgical cases, open procedures are performed more frequently than laparoscopic approaches, resulting in extended hospital stays and increased mortality rates.

Second, manual reduction can alleviate intestinal obstruction, thereby increasing available intraperitoneal space and facilitating laparoscopic surgery. Laparoscopic surgery, compared with open surgery, results in reduced blood loss, shorter hospital stays, and fewer postoperative complications (8–10). Additionally, laparoscopic surgery enables the assessment of an obturator hernia on the contralateral side and facilitates the detection of any concomitant hernias.

Third, successful elective surgery following manual reduction facilitates a cleaner surgical field and enhances the feasibility of employing artificial materials, such as mesh and plugs. Non-mesh repairs are associated with a significantly higher recurrence rate than mesh repairs (10%) (7), indicating that surgical procedures using artificial materials are preferable. At our institution, to minimize surgical invasiveness, we employ a straightforward technique that requires minimal peritoneal dissection and involves the insertion of a plug into the obturator foramen (3). In this study, laparoscopic surgery was completed in 12 cases, with plug-assisted procedures performed in 11 of these cases. Obturator hernias frequently occur in elderly patients, a population often characterized by increased frailty. Careful management is required to minimize the risk of complications. In this study, antithrombotic medication was not suspended in patients with cerebral infarction or ischemic heart disease undergoing antithrombotic therapy. However, due to the risk of postoperative wound bleeding and pelvic hematoma, suspension of medication might be required based on individual patient risk and clinical circumstances.

If manual reduction is successful, the following points should be considered.

First, the risk of intestinal perforation must be considered. Reportedly, the risk of perforation increases 72 h after the onset of an obturator hernia (2). Therefore, in cases with delayed presentation, open surgery should be considered with particular caution. In this study, cases in which reduction was performed 4 days (96 h) after symptom onset exhibited intestinal necrosis and perforation. Ongoing monitoring of the patient's overall condition and laboratory parameters is recommended following the reduction. In this study, the median duration from reduction to surgery was 4 days (range 0–9 days). Based on these findings, we believe that surgical repair should be performed as soon as possible after reduction of an incarcerated obturator hernia to minimize the risk of perforation and recurrence.

Second, as many patients are elderly and have multiple comorbidities, surgical intervention should be considered in light of the patient's life expectancy and overall health status. We encountered a case in which a patient died shortly after the reduction due to advanced age. Given that IOH frequently occurs in elderly individuals, the decision to perform elective surgery requires careful consideration of the patient's overall health status, comorbidities, and life expectancy.

A major limitation of this study is the absence of a comparison group, such as patients who underwent immediate surgery without manual reduction. As a result, it is difficult to definitively assess the degree to which manual reduction provides a clinical benefit.

## 5 Conclusion

Laparoscopic surgery after manual reduction of IOH represents an effective and minimally invasive therapeutic strategy, demonstrating a relatively high rate of success. However, as many patients with obturator hernias are elderly and frail with multiple comorbidities, and because the small intestine may become incarcerated, leading to necrosis and perforation, early diagnosis and intervention are essential. Additionally, continuous and careful evaluation of the patient's overall condition throughout treatment is necessary.

## Data Availability

The raw data supporting the conclusions of this article will be made available by the authors, without undue reservation.
